# Phenomenology, psychiatric comorbidity and family history in referred preschool children with obsessive-compulsive disorder

**DOI:** 10.1186/1753-2000-6-36

**Published:** 2012-11-22

**Authors:** Murat Coskun, Salih Zoroglu, Mucahit Ozturk

**Affiliations:** 1 Istanbul University, Istanbul Medical Faculty, Child and Adolescent Psychiatry Department, Istanbul, Turkey; 2 Istanbul University, Istanbul Medical Faculty, Child and Adolescent Psychiatry Department, Istanbul, Turkey; 3 Center for Psychiatric Research, Training and Consultation (PEDAM), Istanbul, Turkey

**Keywords:** Children, Preschool, Obsessive-compulsive disorder, Comorbidity, Family history

## Abstract

**Objective:**

The study aimed to investigate phenomenology, psychiatric comorbidity, and family history of obsessive-compulsive disorder (OCD) in a clinical sample of normally developing preschool children with OCD.

**Method:**

Subjects in this study were recruited from a clinical sample of preschool children (under 72 months of age) who were referred to a university clinic. Subjects with a normal developmental history and significant impairment related to OCD symptoms were included in the study. Children’s Yale-Brown Obsessive-Compulsive Scale was used to assess OCD symptoms. Each subject was assessed for comorbid DSM-IV psychiatric disorders using a semi-structured interview. Parents were evaluated for lifetime history of OCD in individual sessions.

**Results:**

Fifteen boys and ten girls (age range: 28 to 69 months; 54.12±9.08 months) were included. Mean age of onset of OCD was 35.64±13.42 months. All subjects received at least one comorbid diagnosis. The most frequent comorbid disorders were non-OCD anxiety disorders (n=17; 68.0%), attention-deficit hyperactivity disorder (ADHD) (n=15; 60.0%), oppositional defiant disorder (ODD) (n=12; 48.0%), and tic disorders (n=6; 24.0%). Mean number of comorbid disorders was 3.65 and 2.35 for boys and girls, respectively. At least one parent received lifetime OCD diagnosis in 68 percent of the subjects.

**Conclusions:**

The results indicated that OCD in referred preschool children is more common in males, highly comorbid with other psychiatric disorders, and associated with high rates of family history of OCD. Given the high rates of comorbidity and family history, OCD should be considered in referred preschool children with disruptive behavior disorders and/or with family history of OCD.

## Introduction

Obsessive-compulsive disorder (OCD) is a relatively common neuropsychiatric disorder with a generally chronic and disabling course affecting up to 3% of young population [1-3]. Previous studies have reported heterogeneity in the age (prepubescent vs. pubertal), type of onset (abrupt vs. insidious, precipitating trigger event vs. none), and course of children's illness (chronic with some fluctuations vs. severe exacerbations with remissions) [4-7]. Most studies noted a male predominance in children with the gender distribution becoming more equal in adolescence [5,7,8]. OCD is a highly comorbid disorder in young population, with up to 80 percent of the subjects meeting diagnostic criteria for another psychiatric disorder, most commonly another anxiety disorder (26–75%), depressive disorder (25–62%), externalizing disorders [18–50%; i.e., attention deficit-hyperactivity disorder (ADHD), oppositional defiant disorder (ODD), conduct disorder (CD)], or tic disorder (15–30%) [8-12]. Meanwhile a higher familial risk among relatives of subjects with early-onset OCD has repeatedly been demonstrated, suggesting that early-onset OCD might be a sub-group of a major genetic etiology [13-17]. In the study by Chabane et al. (2005), 32.6% of the probands with early onset OCD had a positive family history of OCD [17]. The average age of onset of OCD was 9.98 ± 3.2 years in this study. In Nestadt et al.’s study (2000), age at onset of OCD in probands was strongly related to familiality [15]. No case of OCD was detected in the relatives of probands whose age at onset of OCD was 18 years or older [15].

Although it was once thought that OCD did not occur in early childhood, onset of OCD has been documented as early as 2 years of age, with estimates of a mean age of 10 years [6,18,19]. Studies have shown that even though the phenotypic features of OCD are similar in children, adolescents, and adults, some clinical characteristics may show developmental specificity [7,20,21]. Research by Eggar and Angold (2006) on psychiatric disorders in preschool children suggested that psychiatric disorders in this population share many of the same characteristics, including rate and pattern of comorbidities, with psychiatric disorders diagnosed in older children and adolescents [22]. However, studies on particular psychiatric disorders, such as OCD, among preschool children are scarce in the literature. Studies by Garcia et al. (2009) [6] and Coskun and Zoroglu (2009) [19] are rare examples of studies on early childhood OCD that included subjects under 6 years of age. The study by Coskun and Zoroglu (2009) reported six preschool children (age range, 40–61 months; 51.5±8.8 months) presented with distressing and disabling OCD symptoms with the onset ranging from 18 to 54 months of age (32±13.5 months) [19]. The study by Garcia et al. (2009) included 58 subjects with an age range of 4 to 8 years [6]. The mean age of OCD onset was 4.95 years (2 to 6 years), and the mean age of presentation was 6.72 years (4 to 8 years). No male predominance was reported in this study. Overall, 12 percent of children did not meet criteria for other diagnosis and twenty percent reported a first-degree family history of OCD [6]. Another recent study by Nakatani et al. (2011) reported that young subjects with very early onset OCD (onset before 10 years of age) are characterized by a longer duration of illness, higher rates of comorbid tics, more frequent ordering and repeating compulsions, and greater parent-reported psychosocial difficulties. They concluded that very early onset OCD may be associated with different symptoms and comorbidities compared to late onset OCD [23]. Meanwhile, clinical observations and systematic investigations have shown that a subgroup of children with OCD and/or tic disorders experiences the onset and subsequent exacerbations of their symptoms following infections with group A beta-hemolytic streptococci (GABHS). This subgroup has been designated by the acronym PANDAS, standing for pediatric autoimmune neuropsychiatric disorders associated with streptococcal infections. Five clinical characteristics define the PANDAS subgroup: presence of OCD and/or tic disorder, onset occurring between 3 years of age and puberty, sudden onset or abrupt exacerbations, association with neurological abnormalities during exacerbations (adventitious movements or motoric hyperactivity), and the temporal association between symptom exacerbations and GABHS infections [24,25].

The present study aims to investigate phenomenology, psychiatric comorbidity, and family history in referred preschool children with distressing and disabling OCD symptoms. Given the limited literature on OCD in preschool children, we believe that this study would contribute to the literature and help clinicians recognize and systematically assess OCD in this special population. We expect that OCD among clinically referred preschool children would relate to high rates of psychiatric comorbidity and family history of OCD, may be associated with different symptoms, and may cause emotional distress and functional impairment as in school aged children.

## Method

### Participants

Subjects in this study were recruited from a clinical sample of preschool children who were presented or referred to Child and Adolescent Psychiatry Department of Istanbul Medical Faculty at the Istanbul University. They were referred for various behavioral and/or emotional problems. Preschool children presented to the clinic underwent routine clinical assessment, and subjects who had a provisional diagnosis of OCD or OCD like symptoms were referred to the first author (M.C) for further evaluation and research participation. Study subjects were recruited from this population of children according to the inclusion criteria defined below. Primary complaints of parents of these children included distressing symptoms of OCD (n= 10; 40.0%), disruptive and oppositional behaviors (n=6; 24.0%), a combination of OCD and disruptive behaviors (n=6; 24.0%), or other problems (n=3; 12.0%). Subjects were homogeneous in terms of ethnic/racial background. All subjects were Turkish descent. Psychopharmacological treatment for OCD in eleven subjects in this study was reported in two previous studies [19,26].

### Instruments

#### 1-Schedule for Affective Disorders and Schizophrenia for School Age Children-Present and Lifetime Version (K-SADS-PL)

The K-SADS-PL is a semi-structured diagnostic interview designed to assess current and past episodes of psychopathology according to Diagnostic and Statistical Manual-fourth edition (DSM-IV) for psychiatric disorders in school aged children and adolescents [27]. Although it has not been originally designed for preschool children, Birmaher et al. (2009) conducted a psychometric study to assess the reliability of the K-SADS-PL in preschool children aged 2- to 5-year-old (mean age 3.8 ± 1.2 years old). The results suggested that the K-SADS-PL is a reliable instrument to evaluate DSM-IV psychiatric disorders in preschoolers, particularly ADHD, ODD, anxiety disorders, mood disorders, and elimination disorders [28]. Gokler et al. (2004) provided support for the reliability and validity of Turkish version (K-SADS-PL-T) [29].

#### 2-Children’s Yale-Brown Obsessive-Compulsive Scale (CY-BOCS)

CY-BOCS is a 10-item, clinician-completed scale assessing severity of illness over the previous week. The CY-BOCS also includes a symptom checklist of more than 60 symptoms of obsessions and compulsions categorized by the predominant theme involved. Psychometric properties are well documented, and the CYBOCS is considered the gold/criterion standard measure of pediatric OCD [30]. The reliability and efficacy of the Turkish version of CY-BOCS has been shown [31].

### Procedure

During the first clinical interview, subjects were assessed for socio-demographics, reason(s) for referral and main complaints, and developmental/medical histories. Subjects were later assessed for eligibility to be included in the study. Inclusion criteria were a) being at or younger than 72 months of age at the time of first clinical presentation, b) receiving DSM-IV diagnosis of OCD and suffering from significant emotional distress and functional impairment in daily life related to OCD symptoms, c) having a normal developmental history, and d) obtaining parental informed consent for the inclusion in the study. Main exclusion criteria were having a provisional or definitive diagnosis of autism spectrum disorder or mental retardation, which were identified through a detailed clinical interview with developmental history, repeated observations, and developmental test (Denver II).

Diagnosis of ADHD, ODD, OCD, and non-OCD anxiety disorders, elimination disorders, and tics disorders was made using K-SADS-PL-T [29]. K-SADS-PL was administered to parents, mostly mothers. Children were allowed to join interview or spend time in playroom. However, all study subjects were questioned directly regarding their OCD symptoms, emotional distress, and functional impairment related to these symptoms, as well as their perceptions of these symptoms. Diagnosis of other disorders that are not included in K-SADS-PL (such as language & speech disorders, trichotillomania, pica, conversion disorder, depressive adjustment disorder and gender identity disorder) was made according to DSM-IV criteria [32]. The difference in number of comorbid diagnoses between genders was examined using Mann–Whitney U test. CY-BOCS was used to assess baseline symptoms by interviewing parents and teachers and observing the child during follow up visits. Gleason et al. (2007) stated that baseline OCD symptoms in preschool children should be assessed with a systematic measure, such as CY-BOCS [33]. The first author (M.C) administered K-SADS-PL and CY-BOCS. Interviewer completed a formal training in administering K-SADS-PL and used this instrument in several published and not yet published studies. Given the characteristics of study subjects, both instruments were administered in different sessions without any time limit. While CY-BOCS was being administered, a special caution was given to differentiate between developmentally normal ritualistic behaviors and OCD symptoms. We discuss how to differentiate between these two conditions in the discussion section. The symptom checklist part of CY-BOCS was administered to all parents and majority of children. Some children could not be interviewed for several reason, such as small age, hyperactivity, or disruptive behaviors. Few symptoms that were not included in symptom checklist were listed in ‘other’ category in CY-BOCS.

Subjects were further assessed for the age of the onset and course of OCD symptoms, triggering factors prior to the onset of OCD symptoms, and temporal relation between the OCD onset and externalizing symptoms. Using non-structured interviews, mothers and fathers were evaluated in separate sessions to assess the presence of lifetime OCD diagnosis according to DSM-IV criteria. The Faculty Ethics Committee approved the study.

## Results

Subjects were fifteen boys and ten girls with an age range of 28 to 69 months (54.12±9.08), with the male/female ratio of 1.5. None of the subjects received diagnosis of autism spectrum disorders or mental retardation. Table 1 shows clinical characteristics of the subjects.

**Table 1:** Clinical characteristics of the subjects

		
**Gender (Males)**	n=15; 60%	
**Age range**	28 to 69 months; 54.12±9.08	
**Age at onset of OCD symptoms**	18 to 60 months; 35.64±13.42	
**Types of OCD symptoms**	Cleanliness	n=19; 76%
	Ordering/symmetry	n=16; 64%
	Shape/color of the clothes	n=15; 60%
	Smelling people or things or his/her own body parts	n=14; 56%
	Hoarding	n=12; 48%
	Asking for reassurance	n=12; 48%
	Frequent checking	n=8 ; 32%
	Shape of hairs	n=8 ; 32%
	Ritualistic behaviors	n=7 ; 28%
	Fear of contamination with germs	n=5 ; 20%
	Preoccupation with colors of things	n=4 ; 16%
	Touching people or things	n=3 ; 12%
	Fear of doing “bad things”	n=2 ; 8%
	Religious obsessions	n=2 ; 8%
	Sexual obsessions	n=1 ; 4%
	Fear of getting harmed, poisoned or sick	n=1 ; 4%
**Course of OCD symptoms**	Continue-saw tooth	n=12; 48%
	Continue-stable	n=6 ; 24%
	Continue with symptom free period	n=4 ; 16%
	Episodic	n=3 ; 12%
**Comorbid DSM-IV Diagnoses**	ADHD	n=15; 60%
	SAD	n=13; 52%
	ODD	n=12; 48%
	SP	n=7 ; 28%
	Tics	n=6 ; 24%
	Pica	n=4 ; 16%
	SoP	n=3 ; 12%
	Nocturnal enuresis	n=3 ; 12%
	Encopresis	n=2 ; 8%
	GAD	n=2 ; 8%
	Stuttering	n=2 ; 8%
	Delay in expressive language	n=2 ; 8%
	Trichotillomania	n=2 ; 8%
	Articulation disorder	n=1 ; 4%
	DAD	n=1 ; 4%
	CD	n=1 ; 4%
	GID	n=1 ; 4%
**Lifetime family history of OCD**	Present in both parents	n=5 ; 20%
	Present only in the mothers	n=9 ; 36%
	Present only in the fathers	n=3 ; 12%
	Absent in both parents	n=6 ; 24%
	Subsendromal OCD in mothers with data lacking for fathers	n=2 ; 8%

ADHD: Attention deficit hyperactivity disorder; CD: Conversion disorder; DAD: Depressive adjustment disorder; GAD : Generalized anxiety disorder; GID : Gender identity disorder; ODD: Oppositional defiant disorder; SAD: Separation anxiety disorder; SP: Special phobia ; SoP: Social phobia.

### Age of onset, types and course of OCD symptoms

Age of onset of OCD symptoms ranged from 18 to 60 months (35.64±13.42). Lifetime OCD symptoms identified among these subjects included excessive and/or pervasive preoccupation with cleanliness (n=19; 76.0%); ordering and/or symmetry of things (n=16; 64.0%); the size, shape, and color of their clothes (n=15; 60.0%); shape of hairs (n=8; 32.0%); smelling people, things, or his/her own body parts (n=14; 56.0%); hoarding (n=12; 48.0%); asking for reassurance (n=12; 48.0%); frequent checking (n=8; 32.0%); ritualistic behaviors (n=7; 28.0%); fear of contamination with germs (n=5; 20.0%); preoccupation with colors of things (n=4; 16.0%), touching people or things (n=3; 12.0%), fear of doing “bad things” or harming people (n=2; 8.0%); religious obsessions (n=2; 8.0%); sexual obsessions (n=1; 4%); and fear of getting harmed, poisoned, or sick (n=1; 4%). Overall, seven subjects (28.0%) had one or more obsessions and compulsions while remaining 18 subjects had only compulsions (72.0%). Four subjects endorsed/expressed their obsessions during interviews while for remaining three subjects, obsessions were inferred from mothers’ reports. Excessive and/or pervasive preoccupation with cleanliness was the earliest OCD symptom to emerge in majority of subjects (n=15; 60.0%). A clear psychosocial stressor or traumatic event was reported in the week preceding the emergence of OCD symptoms in 4 subjects (16.0%).

The course of OCD symptoms fell into four categories: a) continuous with fluctuating severity and/or symptoms (n=12; 48.0%); b) continuous with the same severity and/or symptoms (n=6; 24.0%); c) continuous with almost symptom free period ranging from one to ten weeks (n=4; 16.0%); d) intense episodes of OCD symptoms lasting three to fourteen weeks, with almost no symptoms between episodes (n=3; 12.0%).

### Psychiatric comorbidity

All subjects received at least one comorbid diagnosis, with six (24.0%) subjects receiving only one comorbid diagnosis and the majority of the subjects receiving multiple comorbid diagnosis. The most frequent comorbid disorders were non-OCD anxiety disorders, attention-deficit hyperactivity disorder (ADHD), and oppositional defiant disorder (ODD). Comorbid DSM-IV diagnoses included ADHD (n=15; 60.0%), separation anxiety disorder (n=13; 52.0%), ODD (n=12; 48.0%), specific phobia (n=7; 28.0%), tic disorder (n=6; 24.0%), pica (n=4; 16.0%), social phobia (n=3; 12.0%), nocturnal enuresis (n=3; 12.0%), encopresis (n=2; 8.0%), generalized anxiety disorder (n=2; 8.0%), stuttering (n=2; 8%), delay in expressive language (n=2; 8.0%), trichotillomania (n=2; 8.0%), articulation disorder (n=1; 4.0%), depressive adjustment disorder (n=1; 4%), conversion disorder (n=1; 4.0%), and gender identity disorder (n=1; 4.0%).

Boys exhibited more comorbid disorders compared to girls (p=0.014, Z= −2.45). The mean number of comorbid disorders was 2.3 and 3.6 for girls and boys, respectively. ADHD was slightly more frequent among boys (n=10; 66.6%) compared to girls (n=5; 50.0%). ODD was also more frequent among boys (n=9; 60.0%) compared to girls (n=3; 30.0%).

Temporal relation between the emergence of OCD and ADHD symptoms varied in subjects with comorbid ADHD diagnosis (n=15). While ADHD symptoms predated OCD symptoms in 11 of the fifteen subjects (73.3%), OCD symptoms predated ADHD symptoms in remaining four subjects (26.7%).

### Family history of OCD

Parents of five subjects (20.0%) had lifetime DSM-IV diagnosis of OCD, mothers of nine subjects (36.0%) had lifetime diagnosis of OCD, and fathers of three subjects (12.0%) had lifetime diagnosis of OCD. Parents of six subjects (24.0%) did not have lifetime diagnosis of OCD. While mothers of two subjects (8.0%) had subsendromal OCD, fathers of these two subjects could not be evaluated due to death or divorce.

## Discussion

### Clinical characteristics of OCD in preschool children

Preschool psychopathology is a recently emerging area that may have important clinical implications in terms of diagnostic and preventive/therapeutic interventions and may contribute to the understanding of developmental psychopathology. Contrary to the previously recognized notion that preschool children cannot develop OCD, recent literature has demonstrated that preschool children can develop and suffer from distressing OCD symptoms [6,19].

Developmentally normal ritualistic behaviors may be similar in form to behavior observed in OCD [18,34]. However, it is important to note that normal rituals enrich socialization and help the child connect to others, whereas obsessions and compulsions are hurtful to the self, and lead to social isolation, withdrawal, regressive, and aggressive behaviors [34]. Behaviors that arise from OCD will cause interference in daily life and distress to the child, family, or environment, whereas similar behaviors that are part of normative development may reflect articulated rules by which the child governs his own behavior or that of others. In line with this consideration, participants had to be significantly affected and impaired by OCD symptoms. Meanwhile, in terms of clinical practice, common presence of comorbid psychopathology and family history of OCD could also be helpful in differentiating between normal ritualistic behaviors and behaviors observed in the context of OCD in preschool children.

All subjects in this study suffered from significant distress and functional impairment in social and family lives related to OCD symptoms. It may be important to note that OCD symptoms were among the primary reason(s) for referral or main concern(s) of the parents in most subjects. For the remaining subjects, distressing symptoms of OCD were determined through clinical evaluation. Parents admitted to using physical punishment in more than half of the subjects to change the child’s behaviors, which were in fact related to OCD. Impairment due to OCD symptoms was differentiated from impairment due to other emotional/behavioral disorders through detailed clinical examination and based on history as well as repeated interviews and observations.

During the most severe and intense period of symptoms, subjects spend a considerable amount of time, ranging from few hours to almost all day, showing OCD symptoms. Moreover, emotional distress and functional impairment related to OCD were not limited to daytime. Some of the subjects also suffered from OCD during night. For instance, a 49-month-old boy was frequently awaking at night and insisting on washing his hands. When the mother asked for the reason, he sometimes reported that he was just doing dirty things, like playing in the sand, and his hands became dirty. He spent more than 4 hours each day cleaning his toys with large amounts of tissue paper and arranging things at home. He would carefully check glasses, spoons, and plates for any spots and refuse to use them if he saw even a small spot. He would take off all his clothes before he would use the toilet and insisted on bathing or wanting to use excessive amounts of perfume after using the toilet to avoid smelling bad. He was smelling people when they wanted to love or touch or play with him and refusing them if he disliked their smell. He was also smelling his hands after touching something such as doors outside at home, his toys or touching somewhere at public transportations. Another girl, 58 month-old age, was awaking at night and insisting that her mother clean her hands with lots of tissue paper because her hands got dried, dirty, oily, or sticky. She was also asking the same questions as she asked during the day. Most subjects also had significant problems with peer and adult relationships. While a group of them refused other children or people coming to their home, as they would make his/her home dirty, another group of them were smelling people, refusing to touch them and play or stay with him/her. Some of them spent great amount of time cleaning his/her toys rather than playing with them.

While making OCD diagnosis, DSM-IV requires that individuals at some point recognize that the obsessions or compulsions are excessive or unreasonable. However, DSM-IV also notes that this does not apply to children. Despite significant distress and impairment related to OCD symptoms, most preschool children in this study were not able to comprehend or verbalize that these symptoms were distressing, excessive, or unreasonable. For instance, one child with obsessional thoughts (69 month-old boy) was partially able to admit, on direct questioning, that his thought of harming his friends but not contamination by germs may be a silly thought that belonged to another person inside of him. Of those with significant compulsions at the time of evaluation (n=20), only six were able to admit and verbalize, on direct questioning, that some of his/her behaviors were distressful for him/herself or their friends and families. These data may suggest that preschool children with OCD may be able to admit and verbalize distressing and excessive nature of their obsessions or compulsions if they were asked about these symptoms directly.

The study by Garcia et al. (2009) [6] included subjects referred to a Pediatric Anxiety Research Clinic. The mean age of OCD onset was 4.95 years and the mean age of presentation was 6.72 years. No male predominance was reported in their study [6]. The current study was conducted in a child and adolescent psychiatry department of a faculty hospital. Because preschool children, particularly males, were more frequently referred for the comorbid externalizing disorders, a male predominance and higher rates of ADHD or ODD could have been expected in our clinical sample. Male/female ratio was found as 3:2 in our study which is generally consistent with the available literature on pediatric OCD (Masi et al. 2005 : 69% male, p = 0.54; Mancebo et al. 2008: 67% male, p = 0.70; Garcia et al. 2009: 39% male, p = 0.12; Nakatani et al. 2001: 58% male, p = 0.98) [5-7,17,21,23,35]. Distressing and impairing OCD symptoms emerged as early as 18 months of age, with most subjects experiencing the onset before three years of age. The mean age of the onset of OCD symptoms was 35.64 months. The course of the illness was generally chronic with stable (24.0%) or more commonly fluctuating (48.0%) severity and/or nature of OCD symptoms. Parents usually defined cleanliness, ordering/symmetry, and smelling compulsions as the most distressing symptoms. In the study by Garcia et al. (2009) [6], 75 percent of the sample endorsed multiple obsessions, particularly contamination and aggressive/catastrophic obsessions. However, seven subjects in our sample (28.0%) had at least one obsessional thought during the course of the illness. This study reported lower frequency of obsessions (28.0%) compared to the ratio reported in the study by Garcia et al. (2009) [6]. The higher mean age of the onset (4.95 years) and presentation (6.72 years) in the study by Garcia et al. (2009) compared to lower mean age of onset (3 years) and presentation (4.5 years) in this study may account for this difference. In addition, the younger the children are, the more difficult it is to observe obsessions even if they are present. It may be important to note that psychiatric examination detected obsessional thoughts, which most parents also recognized as distressing, excessive, and unreasonable. An interesting example concerns a 62-month-old girl with severe religious and sexual obsessions accompanying other OCD symptoms. She frequently expressed her fears of blasphemy with “I just had a bad thought about the God”, “I thought a sinful thing”, “Will the God punish me?”, “The God will get angry at me”, “But I love the God”. She frequently asked her mother for reassurance about her actions while she was crying. She was also suffered additional, seriously distressing sexual obsessions. The mother expressed her concern about child thinking of teacher’s penis and becoming distressed with seeing her teacher’s chest through his shirt’s collar. She asked too many questions, such as “I saw my teacher’s chest. Would I make shameful thing with him?”. The youngest subject with obsessions was a 28 month-old girl suffering contamination obsession for two months. She expressed her fear and distress of being contaminated by germs if she approached wastebasket, played on the ground, or touched her toys without cleaning them.

### Psychiatric comorbidity & family history

Childhood OCD is frequently comorbid with other psychiatric disorders, particularly with disruptive behavior, anxiety, mood, and tic disorders [8-12]. Retrospective studies suggest that disruptive behavior symptoms may predate the onset of OCD [9,36]. For example, Geller et al. (1996) found that 33% of a sample of juvenile OCD patients also met criteria for ADHD and 43% met criteria for ODD [9]. However, the mean ages of onset were before age 2 years for ADHD and age 7.1 years for ODD, hence typically predating the onset of OCD at mean age of 8.5 years. Geller et al. (2001) further reported that regardless of the age of assessment, younger age of OCD onset was associated with higher rates of ADHD and non-OCD anxiety disorders [37]. The study by Garcia et al. (2009) on early childhood OCD reported that children with comorbid ODD had an earlier age of OCD onset compared to children without ODD [6]. However, most studies on childhood OCD did not report specific findings on the psychiatric comorbidity regarding the gender. Garcia et al. (2009) reported no gender differences in the presence or number of comorbid conditions [6].

Regarding comorbidity, our study reported both similar and different findings compared to previous studies of childhood OCD. As expected, the results revealed high psychiatric comorbidity, with all subjects having at least one comorbid disorder. Non-OCD anxiety disorders (68%), externalizing disorders, such as ADHD (60%) and ODD (48%), and tic disorders (24%) were among the most frequent comorbid disorders. These findings are consistent with the previous report that, regardless of the age of assessment, younger age of onset was associated with higher rates of ADHD and non-OCD anxiety disorders [37]. However, while ADHD symptoms predated OCD symptoms in 11 out of the fifteen subjects (73.3%), OCD symptoms predated ADHD symptoms in remaining four subjects (26.7%). This is somewhat different from previous findings, which reported that externalizing symptoms significantly and consistently predated OCD symptoms [9]. In the study by Garcia et al. (2009), 12 percent of children did not meet the criteria for any diagnosis other than OCD, and they reported no differences between boys and girls in the presence or number of comorbid conditions [6]. However, we found higher rates of comorbid disorders, with boys exhibiting more comorbid disorders compared to girls. The mean number of comorbid disorders was 3.6 and 2.3 for boys and girls, respectively. ADHD and particularly ODD were more frequent among boys compared to girls. However, these findings may be of limited value because small sample size did not allow us to examine this relationship accurately. In addition, the main reason for different findings in this study may be the fact that this study included clinically referred preschool children with possible multiple psychiatric disorders and significant impairment that may not be reflected in a community sample. Generally, early onset psychiatric disorders, such as early onset schizophrenia or bipolar disorder, are more severe compared to adult onset disorders, and they may be associated with more frequent comorbid psychopathology [38,39]. However, because we did not include control group, we were not able to examine the relationship among very early onset, severity of illness, and psychiatric comorbidity in our sample. Further research is needed to determine whether very early onset OCD relates to more severe course or higher rates of comorbid psychopathology.

Another comorbid condition that may merit discussion is Pica. Although etiology of Pica is not fully understood, some authors [40-42] describe an association between pica and obsessive–compulsive spectrum disorder (OCSD). It has also been reported that adult and young patients with pica symptoms responded to SSRI treatment [41,42]. Relatively high rate of pica in this sample (16%) may provide further evidence for an association between pica and OCSD. One of the subjects in this study (63-months old girl) showed significant improvement in pica following an escitalopram treatment for both OCD symptoms and pica.

A high ratio of family history of OCD, up to 32 percent, has been reported in young subjects with OCD [17], and the age at onset of OCD in probands related strongly to familiality [15]. In the study by Garcia et al. (2009), twenty percent of the subjects reported a first-degree family history of OCD [6]. The current study found a higher rate of family history of OCD. At least one parent received lifetime OCD diagnosis in 68 percent of probands. This is two to three times higher than previously reported rates. The lower age at the onset, 35 months, in this study compared to previous studies may have contributed to higher rates of family history. However, an ascertainment bias may have also contributed to this finding. It is important to note that more than half of the subjects were referred for OCD symptoms. Thus, most parents were familiar with OCD as a psychiatric disorder from their own past or current experience. Consequently, they were able to recognize the symptoms more easily and seek help for problematic behaviors related to OCD in their child. This may as also reflect high rates of family history of OCD among our sample.

### Conclusions & limitations

The current study is the second attempt, to our knowledge, to investigate phenomenology, psychiatric comorbidity, and family history of OCD in preschool children with OCD. The results of this study may contribute to the understanding of OCD as a neurodevelopmental disorder across the lifespan. The major findings of this study are that OCD may emerge in preschool children as early as at two years of age, and it seems to be more common in males. It has generally a chronic course with alternating severity and/or symptoms. It is frequently comorbid with other psychiatric disorders, particularly non-OCD anxiety disorders, disruptive behavior disorders, and tic disorders. OCD in referred preschool children might relate to high rates of family history of OCD. These findings may have important clinical implications, suggesting that referred preschool children with disruptive behaviors and anxiety symptoms should be screened for OCD, particularly in the presence of family history of OCD.

This study has several methodological limitations that should be addressed. Diagnosis of major psychiatric disorders in this very young population was made using K-SADS-PL, which was not originally designed for preschool children. However, no other instruments in our country exist to assess psychopathology in preschool children; moreover, evidence suggests that K-SADS-PL can be used to assess preschool psychopathology [6,28]. Lack of a structured interview in parental diagnosis is another limitation. It should be noted that findings of this study cannot be generalized, as the study included a relatively small sample consisting of clinically referred subjects with significant impairment. In addition, we did not include control group to compare rates of comorbidity and family history. As we discussed above, including clinically referred subjects with significant impairment can be considered an ascertainment bias that contributed to the high rates of psychiatric comorbidity and family history of OCD and a male predominance within the sample. More systematic research on this area should be conducted with larger samples and with the inclusion of control groups.

## Competing interests

The author(s) declare that they have no competing interests.

## Authors’ contribution

All authors (MC, SZ and MO) contributed to study design, literature review, manuscript writing, and final approval. M.C conducted clinical interviews and follow-up of the study subjects and wrote the first draft of the manuscript. S.Z and M.O reviewed manuscript and approved its final form.
